# Designing Dual Inhibitors of Autotaxin-LPAR GPCR Axis

**DOI:** 10.3390/molecules27175487

**Published:** 2022-08-26

**Authors:** Souvik Banerjee, Suechin Lee, Derek D. Norman, Gabor J. Tigyi

**Affiliations:** 1 Department of Chemistry, Middle Tennessee State University, 1301 E. Main Street, Murfreesboro, TN 37132, USA; 2 Molecular Biosciences Program, Middle Tennessee State University, 1301 E. Main Street, Murfreesboro, TN 37132, USA; 3 Department of Physiology, University of Tennessee Health Science Center Memphis, 3 N. Dunlap Street, Memphis, TN 38163, USA

**Keywords:** autotaxin (ATX), lysophosphatidic acid (LPA), lysophosphatidic acid receptor subtype-1 (LPAR1), dual inhibitors, combination therapy, cancer, metastasis, idiopathic pulmonary fibrosis (IPF)

## Abstract

The ATX-LPA-LPAR1 signaling pathway plays a universal role in stimulating diverse cellular responses, including cell proliferation, migration, survival, and invasion in almost every cell type. The ATX-LPAR1 axis is linked to several metabolic and inflammatory diseases including cancer, fibrosis, and rheumatoid arthritis. Numerous selective ATX or LPAR1 inhibitors have been developed and so far, their clinical efficacy has only been evaluated in idiopathic pulmonary fibrosis. None of the ATX and LPAR1 inhibitors have advanced to clinical trials for cancer and rheumatoid arthritis. Nonetheless, several research groups, including ours, have shown considerable benefit of simultaneous ATX and LPAR1 inhibition through combination therapy. Recent research suggests that dual-targeting therapies are superior to combination therapies that use two selective inhibitors. However, limited reports are available on ATX-LPAR1 dual inhibitors, potentially due to co-expression of multiple different LPARs with close structural similarities at the same target. In this review, we discuss rational design and future directions of dual ATX-LPAR1 inhibitors.

## 1. Introduction

**Figure 1:**
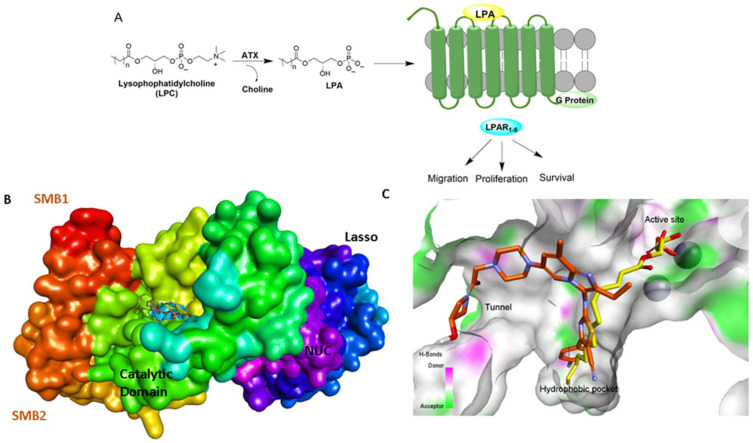
ATX-LPA-LPAR signaling axis and ATX binding sites. (**A**). ATX catalyzes the formation of LPA from LPC and subsequently activates LPARs. (**B**). Depiction of ATX surface and domains (PDB: **3NKN**). Abbreviations used: SMB, somatomedin-like domain; NUC, nucleotidase-like domain. (**C**). ATX tripartite binding site with GLPG 1690 (red) and LPC (yellow).

Ectonucleotide pyrophosphatase 2 (ENPP2) commonly referred to as Autotaxin (ATX) was initially discovered through its lysophospholipase D activity [1]. ATX hydrolyzes extracellular lysophospholipids, primarily lysophosphatidylcholine (LPC) due to its high abundance in biological fluids, to generate the lipid mediator lysophosphatidic acid (LPA) [2,3]. LPA induces several cellular responses, including cell proliferation, migration, survival, invasion, metastasis, and production of cytokines, by modulating six distinct LPA G-protein coupled receptors (LPAR) (Figure 1) [1,2,3,4,5,6]. Activation of the ATX-LPA-LPAR signaling pathway is critical for the maintenance of stemness in pluripotent somatic and cancer stem cells [7,8], the development of neurological systems, the generation of blood vessels, wound healing, and tissue repair [4,5]. Consequently, dysregulation of this signaling pathway is often linked to metabolic and inflammatory disorders, including cancer, tumor immunity [9,10,11,12], fibrosis, neuropathic pain and neurodegeneration [13,14,15], inflammation, autoimmune diseases, and metabolic syndrome [4,5,16]. In this context, genetic studies have demonstrated a decisive role of LPAR1 and ATX in mouse models of lung fibrosis [3,16,17,18,19,20]. A study of idiopathic pulmonary fibrosis (IPF) patients has indicated aberrant levels of LPA in the bronchoalveolar lavage (BAL) fluid [21]. Overexpression of ATX has also been documented in patients with IPF [17]. Apart from IPF, extensive research has shown that blocking the ATX-LPA-LPAR axis can have promising therapeutic benefits on a variety of illnesses, including cancers of the breast, ovary, pancreas and liver, cardiovascular diseases, pregnancy induced hypertension (preeclamsia), inflammation induced systemic bone loss, rheumatoid arthritis, and suppression of antitumor immunity [1,2,3,4,5,16,17,21,22,23,24,25,26]. Therefore, there has been extensive interest in developing new therapeutics targeting the ATX-LPAR signaling pathway [1,2,3,22,27]. In concert with these efforts, Katsifa et al. have demonstrated that long-term pharmacologic suppression and widespread genetic deletion of ATX in adult mice are well tolerated, allaying possible toxicity concerns of ATX therapeutic targeting [28]. In addition, Lin et al. have demonstrated that genetic deletion of ATX in mice suppresses experimental colitis [29]. This review discusses recent advances in ATX inhibitors and the advantages of simultaneous inhibition of ATX and LPAR1.

## 2. Structural Aspects of ATX Inhibitors

**Figure 2:**
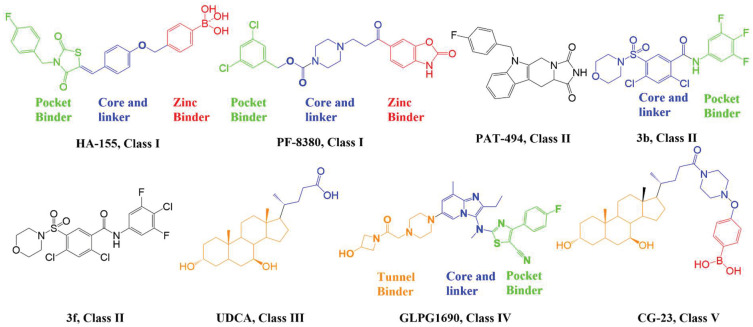
Classification of recently developed non-lipid small molecule ATX inhibitors. 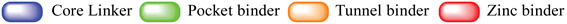.

Lipid-like and non-lipid ATX inhibitors have been reported over the last decade, with co-crystal structures revealed for inhibitors LPA (14:1, PDB: **3NKN**, Figure 1) and HA-155 (PDB: **2XRG**, Figure 2) [30,31]. ATX inhibitors can be divided into five different classes based on their binding mode, namely: class I, orthosteric site; class II, hydrophobic pocket; class III, allosteric tunnel; class IV, tunnel-pocket hybrids; and class V, tunnel-active site hybrids, which exhibit a distinct binding mode in the tripartite interacting site in the T-shaped binding pocket of ATX (Figure 1) [4,32]. A significant number of these inhibitors, including HA-155 and PF-8380 (PDB: **5OLB**, Figure 2), belong to the category of orthosteric-site inhibitors as their mode of action is to completely block the substrate by binding to the active site and the hydrophobic pocket [32,33]. Hydrophobic pocket ATX inhibitors have been designed to compete with substrate binding without requiring the headgroup aimed for the active site, such as PAT-494 (PDB: 4ZGA, Figure 2) [4,34]. Our group has reported several potent small molecule non-lipid hydrophobic pocket ATX inhibitors, such as **3b** and **3f** (Figure 2), that exhibit efficacy in vivo [1,27,33]. ATX tunnel binders such as UDCA (Figure 2) demonstrate moderate efficacy due to their non-competitive binding to the hydrophobic tunnel [32]. A recent expansion approach had led to the development of potent tunnel-pocket hybrid inhibitors such as GLPG1690 (PDB: **5MHP**, Figure 2) [2,4,35]. The latest efforts have provided access to the first tunnel-active site hybrid by combining important structural motifs of ATX tunnel binders with that of ATX orthosteric inhibitors (PDB: **7Z0N**, Figure 2) [32].

## 3. ATX-LPAR1 GPCR Axis in Idiopathic Pulmonary Fibrosis and Cancer

Despite significant progress in the development of these different classes of ATX inhibitors, only five drug candidates that target the ATX-LPAR signaling pathway have been evaluated in clinical trials [4,5]. The pocket- and tunnel-binding hybrid GLPG1690 reached a Phase-III clinical trial for the treatment of IPF before the trial was halted in the developmental stage [4]. According to reports, patients taking GLPG1690 for at least six to nine months exhibit a higher mortality risk than patients taking a placebo [35]. Hence, the trial was discontinued in the late stage as the risk-benefit profile was no longer favorable. Currently, the orally active non-competitive ATX inhibitor cudetaxestat (BLD-0409, compound 10, Table 1), which binds in the ATX tunnel, is in a phase II clinical trial for the treatment of IPF (NCT05373914) [36,37]. BLD-0409 shows significant reduction in lung fibrosis, fibrotic markers, and levels of profibrotic LPA in pulmonary fibrotic mouse models [38]. BLD-0409 blocks ATX in a dose-dependent manner, unlike the linear effects of GLPG1690 [4,21]. It is noteworthy that the pocket-tunnel binding compound GLPG1690 competes with the ATX substrate LPC for the hydrophobic pocket region. As LPC occupies the pocket and active site (Figure 1), it is believed that the efficacy of GLPG1690 may be limited when LPC is present at higher concentrations [37,38]. Unlike GLPG1690, BLD-0490 blocks ATX by binding to the allosteric site without interacting with the substrate and may retain its potency at elevated levels of LPC. Due to aberrantly high levels of LPA and overexpression of LPAR1 in IPF patients, LPAR1 inhibitor such as BMS-986020 have also been evaluated in clinical trials for the treatment of IPF [39,40]. A 26-week clinical trial demonstrated that treatment of IPF patients with BMS-986020 results in significant improvement in forced vital capacity (NCT01766817) [39,41]. However, hepatobiliary toxicity has also been reported in some patients receiving BMS-986020. Consequently, a second-generation LPAR1 inhibitor, BMS-986278, was developed and results from phase I studies have shown no evidence of hepatobiliary toxicities. BMS-986278 is currently being evaluated in phase II clinical trials (NCT04308681) for the treatment of IPF [19]. Lastly, the selective oral LPAR1 antagonist SAR100842 was evaluated in clinical trials for their safety and efficacy in reducing skin sclerosis in patients with diffuse cutaneous systemic sclerosis (NCT01651143) [42].

In the context of cancer, research findings over the last decade suggest that two factors, namely (i) resistance to chemo- and radiation-therapy and (ii) the role of the tumor microenvironment (TME) represent the greatest obstacles to inhibition of cancer progression and therapeutic efficacy [5]. A major barrier to therapeutic interventions lies in the existence of cancer stem cells (CSC), whose slower rates of proliferation, self-renewal capabilities, and upregulation of drug efflux transporters contribute to therapy resistance [5,43]. Growing evidence suggests that LPA activates CSC-associated genes, including stem-cell surface markers, antioxidants, and drug transporters [5]. The ATX-LPAR axis is also upregulated in CSC and the inhibition of ATX and LPAR1 attenuates the CSC-like characteristics of ovarian and breast cancers [1,5,8,44]. These observations are corroborated by reports that established the critical role of LPA in maintaining stemness [45,46,47]. In addition to earlier reports demonstrating a fundamental role in tumorigenesis and epithelial-mesenchymal transition [48], recent findings suggest that the ATX-LPAR axis plays a decisive role in influencing cells in the TME. The inhibitory action of LPA in blocking T cell receptor activation, thereby reducing tumor immunity, has been clearly established [9,10,11,12]. Consequently, there is increasing interest in developing new ATX and LPAR1 inhibitors to suppress cancer progression. Efforts to identify new candidates targeting the ATX-LPAR axis received a significant boost from the solving of the crystal structure of LPAR1 co-crystalized with an inhibitor [49].

## 4. Progress with Different Classes of ATX Inhibitors

### 4.1. Orthosteric Inhibitors (Class I)

Lipid-like ATX inhibitors that primarily belong to class I have achieved limited success in preclinical and regulatory development as a result of their high partition coefficient (logP > 5), making them potentially incompatible with clinical development [1,4,27,33]. Discovery of the ATX inhibitor co-crystal structure has provided insights into the active site surfaces and many research groups have used this structure to design small molecule non-lipid class-I ATX inhibitors, such as HA155 and PF-8380 (Figure 1) [50,51,52,53,54]. These inhibitors consist of a core spacer, a hydrophobic tail to occupy the hydrophobic pocket, and a carboxylic acid or its bioisostere to interact with zinc ions located in the active site. However, none of the orthosteric (class I) inhibitors have successfully completed advanced clinical trials, potentially due to off target effects. Hence, recent approaches have focused on developing non-carboxylic acid, non-lipid ATX inhibitors targeting the hydrophobic pocket (class II), the tunnel (class III, allosteric site), or pocket-tunnel hybrid sites (class IV).

### 4.2. Hydrophobic Pocket Inhibitors (Class II)

Hydrophobic pocket inhibitors (class II) obstruct binding of the LPC substrate to ATX [1,4,33]. To the best of our knowledge, our group was the first to report a hydrophobic pocket ATX inhibitor (**1**, Table 1), obtained through virtual and high-throughput screening [33]. The benzamide head group of these compounds reaches deep into the hydrophobic pocket and the morpholine ring sits at the entrance of the tunnel. We have performed structure-activity relationship (SAR) optimization (2–5, Table 1) to improve in vitro and in vivo efficacies of this class of compounds [1,27]. These compounds significantly inhibit melanoma metastasis into the lungs and re-sensitize taxol-resistant breast CSC to paclitaxel [1]. Pantsar et al. (2017) performed a virtual screening and biological evaluation to identify a pyranopyrazole derivative (6, Table 1) of (*S*) conformation that exhibits high potency inhibition of ATX [55]. Molecular docking experiments suggest that the 3,4-dichlorophenyl head group enters the hydrophobic pocket, the 6-amino-dihydropyranopyrazole-5-carbonitrile moiety acts as the linker, and the 4-chlorobenzyloxy group reaches closer to the tunnel. Zhai and colleagues (2018, 2020) reported dihydropyridopyrimidine carbohydrazide as potent ATX-EGFR dual inhibitors (7 and 8, Table 1) [56,57]. These inhibitors fit primarily deep into the hydrophobic pocket and the other building block extends closer to the tunnel.

### 4.3. Allosteric Tunnel Inhibitors (Class III)

Allosteric tunnel binders deter the release and transport of hydrolyzed LPA [4]. This class of inhibitors represents non-competitive ATX inhibitors that fail to compete with the LPC substrate [58]. Miller et al. (2017) reported that the highly potent N-substituted indole derivatives (9 and 10, Table 1) bind at the allosteric site (tunnel), away from the catalytic site [58]. The optimized compound with N-pyrazole substitution (10, Table 1) exhibits good water solubility and lipophilic ligand efficiency (LLE) [58].

### 4.4. Pocket-Tunnel Hybrids (Class IV)

Shah et al. (2016) used HTS to identify imidazopyridine derivatives and performed hit-to-lead synthetic optimization to obtain a potent compound (11, Table 1) [59]. Analysis of X-ray cocrystal structures revealed that the trifluoromethoxy benzene group penetrates deep into the hydrophobic pocket and the (*S*)-4-chlorophenylethylamine group interacts with the tunnel. The (*S*)-confirmation is very important, as it enhances potency manifolds. Recently Zhai and colleagues (2020, 2022) reported potent class IV derivatives of GLPG1690 with flexible carbamate and urea moieties that combine important structural motifs of GLPG1690 and PF-8380 (12 and 13, Table 1) [2,3]. These inhibitors demonstrate excellent antitumor efficacy against Hep3B and RAW264.7 cell lines that are known to overexpress ATX mRNA. Recently, Ma et al. (2021) reported the BIO-32546 compound (14, Table 1), which exhibits ATX inhibitory efficacy in the low nanomolar range [22]. X-crystallographic analysis suggests that these classes of compounds belong to pocket-tunnel hybrid (class IV), since they occupy the pocket with a 4-trifluoromethylcyclohexyloxy moiety and the tunnel with an 8-azabicyclo [3.2.1] octane-3-carboxylic acid moiety.

### 4.5. Tunnel-Pocket Hybrid Inhibitors (Class V)

Clark et al. (2022) recently introduced tunnel-pocket hybrid inhibitors (class V) that combine important structural motifs of non-competitive tunnel binders and competitive orthosteric inhibitors (PDB: 7Z0N) [32]. These inhibitors occupy the tunnel and catalytic site and behave in a competitive manner with the LPC substrate. This series of partially orthosteric-allosteric inhibitors did not demonstrate interactions with the catalytic site. The indirect inhibition of LPAR1 internalization was accomplished by these inhibitors through modulation of LPA-mediated ATX allostery.

It has now been accepted by a large part of the scientific community that inhibitors in Classes II, III, and IV more effectively block the effects of ATX than do those in Class I. These binding modes also allow design and development of more selective ATX inhibitors due to their distance from the zinc ion-containing active site.

## 5. Biological Prospective of ATX-LPAR1 Dual Inhibition

LPA exerts growth factor-like effects in the majority of cell types. Different LPA species can activate six LPARs to regulate a range of biological responses, including cell proliferation, survival, migration, and invasion. LPA is generated either by hydrolysis of LPC by ATX or via the de novo pathway of glycerol 3-phosphate esterification by glycerol-3-phosphtae acyltransfarase-1 (GPAT-1). ATX, LPAR1 and LPAR2 are overexpressed in different tumor types, including ovarian cancer, osteocarcinoma, metastatic melanoma, neuroblastoma, breast cancer, pancreatic cancer, prostate cancer, and hepatocellular carcinoma [43,60]. Expression of ATX and/or LPAR can be further increased upon chemo- or radiotherapy, as shown for breast cancer [44,61].

Therapy to inhibit ATX, LPAR1 or LPAR2 significantly reduced the incidence of metastasis and resistance to chemotherapy [5,8]. We showed that a combination therapy with an LPAR1 inhibitor (Ki16425) and an ATX inhibitor (BMP22) is more effective in inhibiting melanoma metastasis than single therapy [62]. Although the expression of ATX and LPAR by cancer cells can dictate tumor progression, we and others found that host/stromal ATX and LPAR in the TME play equally important roles in influencing cancer progression, either directly or through the regulation of tumor immunity [9,10,11,12]. We showed that a knockout (KO) mouse that lacked LPAR1 demonstrated strong resistance to melanoma metastasis, suggesting that stromal LPAR1 modulates B16F10 melanoma metastasis to the lung [62]. Notably, B16F10 melanoma cells express ATX at high levels but do not express LPAR1, indicating that the therapeutic success of combined inhibition of LPAR1 and ATX with Ki16425 and BMP22, respectively, in suppressing melanoma metastasis is due to targeting of the ATX-LPAR axis in both cancer and stromal cells.

**Figure 3:**
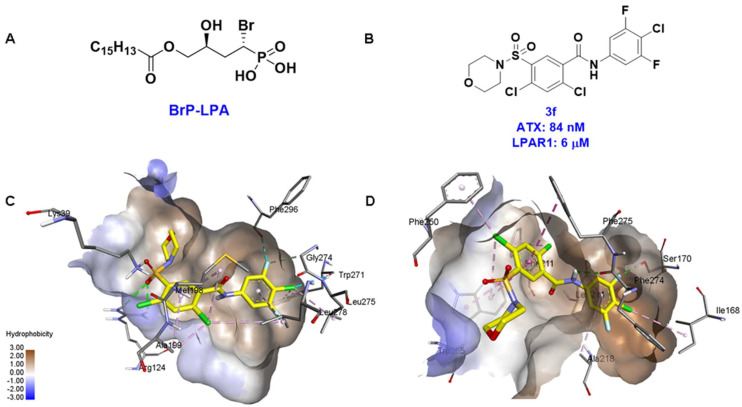
ATX inhibitors with LPAR inhibitory efficacy. (**A**). ATX-LPAR pan antagonist BrP-LPA. (**B**). ATX/LPAR1 dual inhibitor **3f**. (**C**). Mode of binding of 3f in LPAR1 binding site (PDB: **4Z35**). (**D**). Binding mode of **3f** in ATX hydrophobic pocket (PDB: **5MHP**).

These findings are consistent with our previous result in which a lipid-like ATX-LPAR pan-antagonist (BrP-LPA, Figure 3) strongly inhibited B16F10 melanoma metastasis into the lung [63]. Likewise, Peyruchaud and coworkers demonstrated that inhibition of LPAR1 or ATX substantially blocked breast cancer bone metastasis [64,65]. In this context, breast cancer cells that overexpress LPAR1 do not express ATX. Hence, these findings suggest that stromal/host ATX and LPAR1 could be targeted simultaneously to further inhibit tumor progression and metastasis [66]. Iwaki et al. (2020) reported that ATX inhibitors enhance the antitumor efficacy of paclitaxel in a breast cancer model [67]. Similarly, we found that simultaneous inhibition of ATX and LPAR1 with a dual inhibitor increased the sensitivity of breast CSC to paclitaxel [1]. Indeed, blocking the ATX-LPAR1 axis reduces the expression of genes associated with multi-drug resistance transporter and antioxidant [68]. Moreover, studies by Erstad et al. (2017) showed in a rat model of hepatic fibrosis and hepatocellular carcinoma (HCC) that targeting the ATX-LPAR1 axis by inhibiting either ATX or LPAR1 decreased fibrosis and HCC development [69]. 

Apart from cancer, ATX-LPAR1 combination therapy or dual inhibitors of ATX and LPAR1 could also be used to treat IPF, where this signaling pathway has major implications in driving fibrosis. Ninou et al. (2018) showed for the first time that combined ATX-LPAR1 antagonism reduced bleomycin-induced pulmonary fibrosis [17]. Although the effect is minor in comparison to single therapy, it certainly suggests that simultaneous inhibition of ATX and LPAR1 could be a promising therapeutic approach that warrants further investigation. In the case of rheumatoid arthritis (RA), Nikitopoulou et al. observed reduced synovial inflammation and hyperplasia in ATX KO mice [70]. Likewise, Miyabe et al. found that LPAR1 KO mice were protected from RA with significant reduction in synovial inflammation, cartilage damage and bone erosion [71]. Subsequently, two independent groups demonstrated that inhibition of LPAR1 with Ki16425 or inhibition of the ATX-LPAR axis with the ATX-LPAR pan-antagonist Br-LPA reduced the clinical severities of collagen-induced arthritis or RA [72,73]. Altogether, these results encourage implication of ATX-LPAR1 combination therapy or dual inhibitors of ATX-LPAR1 as treatment for various cancers, IPF and rheumatoid arthritis.

## 6. Strategies for Designing Dual Targeting Inhibitors

Solving of the LPAR1-inhibitor cocrystal structure (**PDB: 4Z35**) spurred the development of new LPAR1 based inhibitors and novel ATX inhibitors with LPAR1 inhibitory activity. Our group reported first a lipid-like ATX-LPAR pan-antagonist that significantly reduced tumor volume and blood vessel density in breast cancer patients, relative to paclitaxel [63]. We recently reported a small molecule dual inhibitor of ATX-LPAR1 (**3f**, Figure 3) that is a weak inhibitor of LPAR1. A substantial number of scientific communities believe that dual inhibitors have significant advantages over combination therapy with single inhibitors for a number of reasons, including (**i**) lower risk of drug-drug interactions [74]; (**ii**) predictable PK profiles [75]; (**iii**) lower chance of target-based resistance of dual acting inhibitors than single inhibitors [76]; (**iv**) dual action inhibitors may overcome compensatory mechanisms [77]; and (**v**) development of dual acting inhibitors may require fewer clinical trials [78,79]. Based on these concepts, the development of dual inhibitors has drawn significant attention in both academia and industry over the past decade. In an effort to address the problems with single-target drugs or combination therapy, numerous research groups have worked to develop multitarget therapeutic agents, particularly dual inhibitors [74,76], which are designed to preserve the benefits of combination therapy while avoiding its drawbacks [76]. Due to the greater challenge associated with designing dual inhibitors, increased efforts with multidirectional strategies have been pursued [76]. Primarily three strategies have been extensively employed to develop dual agents, namely (**i**) drug repurposing, (**ii**) active pharmacophore amalgamation, and (**iii**) in silico modeling-based approaches [79]. Drug repurposing seeks to identify new therapeutic indications for known drugs [80]. This process is noted for its cost effectiveness and efficient developmental process with respect to the conventional drug discovery process. A number of dual inhibitors discovered during the drug repurposing process share structural similarities of their binding pockets [81]. Pharmacophore amalgamation has been primarily accomplished using two approaches: pharmacophore linking or pharmacophore merging. The pharmacophore linking approach has gained popularity, as it allows the connection of pivotal pharmacophores from multiple selective parent inhibitors into a single candidate [82]. This is a straightforward strategy to bring together crucial functional groups of two different molecules by introducing a suitable linker. The pharmacophore linking strategy is especially useful for the design of potent dual inhibitors from two selective inhibitors that lack a common pharmacophore. However, this approach often leads to higher molecular weight candidates with potentially adverse bioavailability and physiochemical properties [83] and the linkers can interfere with the interactions between the binding sites and the pharmacophores [76]. In contrast, the pharmacophore merging approach allows us to construct a common feature pharmacophore by extracting overlapping features from different selective inhibitors, thereby resulting in hybrid candidates [80]. The pharmacophore merge strategy requires bioactive molecules that possess similar pharmacophore features, often leading to lower molecular weight candidates with favorable physiochemical properties for further hit-to-lead optimization [82]. However, some structural modifications to the parent active molecule may result in diminished efficacy, necessitating precise identification of pharmacophore characteristics. Recently, computational based approaches have been widely employed to expedite the discovery of dual inhibitors [84,85], Computational approaches, including structure-based or ligand-based drug discovery, are greatly advantageous in the absence of either multiple active inhibitors or active site information of a new target. These computational approaches allow us to perform structure/ligand-based pharmacophores, molecular docking, and molecular dynamics (MD) simulation-based virtual screening to find a candidate with high binding affinity and binding free energy for multiple targets.

## 7. The Ins and Outs of ATX Activity Measurement

In the field, methods for the measurement of ATX lysophospholipase D activity have evolved over time [86]. Initially, thin layer chromatography was used to assess generation of radiolabeled LPA by ATX, using ^14^C-palmitoyl-LPC as a substrate [87]. This technique was later modified to use fluorescently labeled LPC to avoid the use of radiolabeled products [88]. Further, there are several methods that allow for the overall quantification of LPA from biological fluids ranging from immunoassays [89] to mass spectrometric analyses [90,91] as well as MALDI-TOF [92]. These techniques, though effective and able to quantify specific LPA species, generally, lack the capacity for moderate- to high-throughput outputs amenable to screening potential ATX inhibitory compounds. Additionally, global LPA quantification in biological samples is not a direct measure of ATX activity, as other mechanisms for LPA production exist in vivo. As such, LPA quantification is better implemented for downstream characterization rather than identification of ATX inhibitors. Among all of these methods, two standard techniques have emerged by which the lysophospholipase D activity of ATX can be measured via moderate throughput, 96-well platforms. Each assay presents unique advantages and disadvantages.

ATX LPLD activity can be measured indirectly by assessing the production of choline in the cleavage of LPC to LPA in an Amplex Red assay. Utilizing an enzymatic cascade, choline oxidase catalyzes metabolism of the choline byproduct to generate hydrogen peroxide, which subsequently reacts with the Amplex Red reagent in the presence of horseradish peroxidase to generate the fluorescent target resorufin, which that is ultimately quantified [93]. The Amplex Red assay has the advantage of utilizing endogenous LPC substrates of varying chain lengths to assess ATX activity in a more biologically relevant way. However, due to the indirect nature of the assay system, it is impossible to use the Amplex Red assay to determine the mechanism of action of an inhibitor. Additionally, due to the enzymatic cascade involved, it is more difficult to discern whether the potential inhibitory compound acts on ATX itself or one (or more) of the other enzymes involved in the cascade in the absence of additional testing.

ATX LPLD activity can also be measured directly using the fluorescence resonance energy transfer (FRET)-based synthetic LPC analog FS-3 (Echelon Biosciences). In this technique, the fluorophore moiety of the FS-3 substrate is quenched or “silenced” in the native molecule but cleavage by the LPLD activity of ATX separates the fluorophore from the quencher, resulting in detectable fluorescence [94]. By providing a direct readout of ATX LPLD activity, the FS-3 assay is amenable to both moderate-throughput screening of numerous inhibitory compounds and determining inhibitory mechanism of action. However, because FS-3 is a synthetic substrate, the biological implications of ATX LPLD activity determined in this way may be less clear.

Ultimately, when searching for ATX inhibitors, utilization of both assay systems is preferred. FS-3 may be used as an initial screening mechanism, followed by confirmation of activity versus endogenous LPC in the Amplex Red assay. Once validated in the Amplex Red platform, compounds may be further characterized for potency and mechanism of action determination in the FS-3 assay. This two-pronged approach provides the most informative measures of inhibitory capacity while maintaining moderate-throughput workflow.

## 8. Conclusions and Future Directions

The ATX-LPAR1 axis plays a unique role in the pathogenesis and progression of cancer, IPF, and RA. Although there are multiple reports of potent selective inhibitors of ATX or LPAR1, their application in the clinical setting is still at its infancy. The tunnel-pocket hybrid ATX inhibitor GLPG1690 was the only inhibitor in its class to advance to a phase III clinical trial for IPF before being withdrawn, as it was believed to be ineffective at the terminal stage of the disease, which is characterized by higher concentrations of the ATX substrate, LPC. Currently, a non-competitive tunnel binding ATX inhibitor, BLD-0409, is the subject of a phase II clinical trial for the treatment of IPF and its success remains to be determined. A number of LPAR1 inhibitors, including BMS-986278, are in clinical trials for IPF, but further studies are imperative to determine their prolonged efficacy. Notably, ATX stimulates lung epithelial cell migration via both LPA-dependent and -independent pathways [95].

Hence, growing evidence suggests that simultaneous inhibition of ATX and LPAR1 will be significantly advantageous in preventing disease pathogenesis. Several studies have shown that dual inhibitors have substantial benefit over combination therapy with two selective inhibitors. Most importantly, dual inhibitors may circumvent drug-drug interactions and issues with unpredictable PK profiles of two different drugs. Although recent advancement in computer-aided drug development led to the development of diverse dual inhibitors with unique targets, there are limited reports on the use of ATX-LPAR1 dual inhibitors. One of the issues in designing ATX-LPAR1 specific dual inhibitors can be attributed to co-expression of multiple different LPARs with close structural similarities in the same target or stromal cells. Although our group has reported the ATX-LPAR pan-antagonist Br-LPA, specific inhibition of ATX-LPAR1 is preferred due to a comprehensive range of LPA actions. In this regard, we recently developed two small molecule dual inhibitors of ATX-LPAR1 with weak inhibitory activity against LPAR1. Solution of the LPAR1-inhibitor cocrystal structure has stimulated a computational approach that involves virtual screening by structure based-pharmacophore, molecular docking, and MD simulation followed by biological evaluations to find dual inhibitor hits for further synthetic optimizations of therapeutic utilities.

## Figures and Tables

**Table 1:** Potent ATX inhibitors targeting hydrophobic pocket and tunnel.

ID	Structure	Biological Efficacy (IC_50_: nM)	Mode of Binding	Cocrystal Structure (PDB)
1	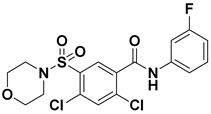	LPC Assay: 43.6 (ATX)	**Pocket binder (Class II)**	N.D.
2	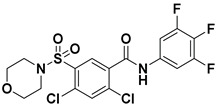	FS-3 Assay: 9 (ATX) 14450 (LPAR1)	**Pocket binder (Class II)** 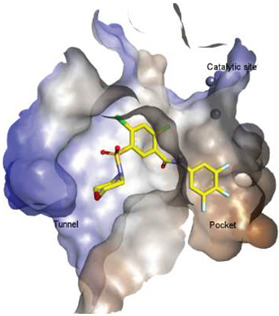	N.D.
3	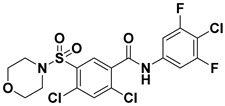	FS-3 Assay: 84 (ATX) 6100 (LPAR1)	**Pocket binder (Class II)**	N.D.
4	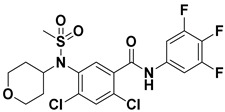	FS-3 Assay: 219 (ATX)	**Pocket binder (Class II)**	N.D.
5	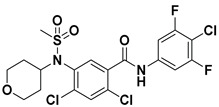	FS-3 Assay: 218 (ATX)	**Pocket binder (Class II)** 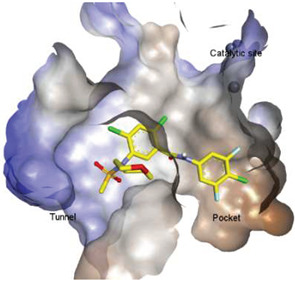	N.D.
6	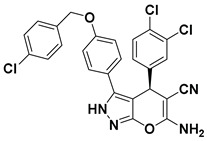	LPC Assay: 87 (ATX)	**Pocket binder (class II)** 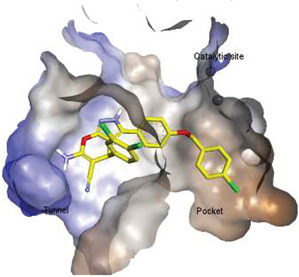	N.D.
7	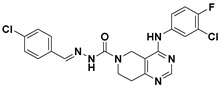	LPC Assay: 24.2 (ATX)	**Pocket Binder (Class II)**	N.D.
8	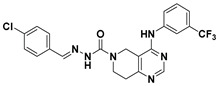	LPC Assay: 15.3 (ATX)	**Pocket Binder (Class II)** 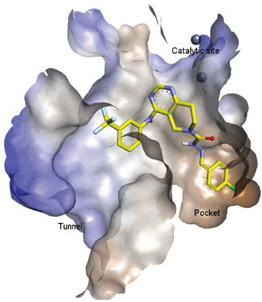	N.D.
9	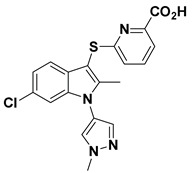	LPC Assay: >300	**Tunnel Binder (Class III)**	**5LQQ**
10	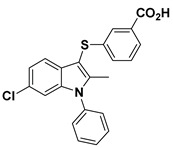	LPC Assay: 81 (ATX)	**Tunnel Binder (Class III)** 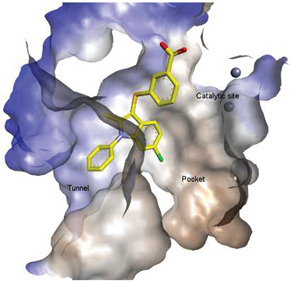	**5LQQ**
11	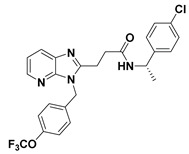	LPC Assay: 1 (ATX)	**Pocket-Tunnel Hybrid (Class IV)** 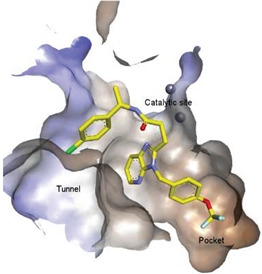	**5LIA**
12	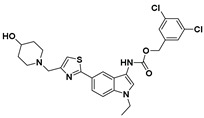	LPC Assay: 1.01 (ATX)	**Pocket-Tunnel Hybrid (Class IV)** 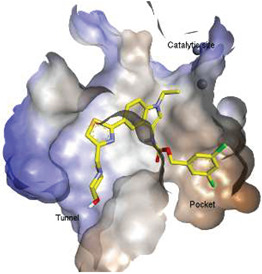	N.D.
13	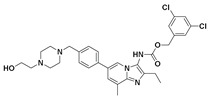	FS-3 Assay: 3.4 (ATX)	**Pocket-Tunnel Hybrid (Class IV)**	N.D.
14	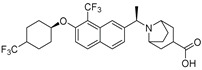	Plasma hlPA: 53 (ATX) ATX FRET: 1 (ATX)	**Pocket-Tunnel Hybrid (Class IV)** 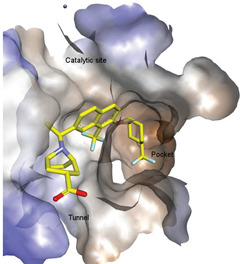	**7MFH**

N.D. Not determined.

## Data Availability

No new data were included or generated in this review.
